# Fecal metagenomic and metabolomic analyses reveal non-invasive biomarkers of *Flavobacterium psychrophilum* infection in ayu (*Plecoglossus altivelis*)

**DOI:** 10.1128/msphere.00301-24

**Published:** 2024-06-17

**Authors:** Mio Takeuchi, Erina Fujiwara-Nagata, Kyohei Kuroda, Kenji Sakata, Takashi Narihiro, Jun Kikuchi

**Affiliations:** 1 Biomedical Research Institute, National Institute of Advanced Industrial Science and Technology (AIST), Ikeda, Osaka, Japan; 2 Department of Fisheries, Kindai University, Nakamachi, Nara, Japan; 3 Bioproduction Research Institute, National Institute of Advanced Industrial Science and Technology (AIST), Sapporo, Hokkaido, Japan; 4 RIKEN Center for Sustainable Resource Science, Yokohama, Kanagawa, Japan; University of Wisconsin-Madison, Madison, Wisconsin, USA

**Keywords:** fecal metabolome, fecal metagenome, non-invasive diagnosis, fish disease, sustainable aquaculture

## Abstract

With the rapid growth of inland aquaculture worldwide, side effects such as the discharge of nutrients and antibiotics pose a threat to the global environments. A sustainable future for aquaculture requires an effective management system, including the early detection of disease through the monitoring of specific biomarkers in aquaculture tanks. To this end, we investigated whether fish feces in aquaculture tanks could be used for non-invasive health monitoring using ayu (*Plecoglossus altivelis*) infected with *Flavobacterium psychrophilum*, which causes bacterial cold-water disease worldwide. Feces that were subsequently produced in the tanks were used for metagenomic and metabolomic analyses. The relative abundances of the genera *Cypionkella* (0.6% ± 1.0%, 0.1% ± 0.2%), *Klebsiella* (11.2% ± 10.0%, 6.2% ± 5.9%), and *F. psychrophilum* (0.5% ± 1.0%, 0.0% ± 0.0%) were significantly higher in the feces of the infection challenge test tanks than in those of the control tanks. The abundances of cortisol, glucose, and acetate in the feces of the infection challenge test tanks were 2.4, 2.4, and 1.3 times higher, respectively, than those of the control tanks. Metagenome analysis suggested that acetate was produced by microbes such as *Cypionkella*. The abundances of indicated microbes or metabolites increased after day 4 of infection at the earliest, and were thus considered possible biomarkers. Our results suggest that feces produced in aquaculture tanks can potentially be used for non-invasive and holistic monitoring of fish diseases in aquaculture systems.

**IMPORTANCE:**

The aquaculture industry is rapidly growing, yet sustainability remains a challenge. One crucial task is to reduce losses due to diseases. Monitoring fish health and detecting diseases early are key to establishing sustainable aquaculture. Using metagenomic and metabolomic analyses, we found that feces of ayu infected with *Flavobacterium psychrophilum* contain various specific biomarkers that increased 4 days post-challenge, at the earliest. Our findings are the first step in establishing a novel, non-invasive, and holistic monitoring method for fish diseases in aquaculture systems, especially in ayu, which is an important freshwater fish species in Asia, promoting a sustainable future.

## INTRODUCTION

Aquaculture is a fast-growing industry; its global food production reached 122.6 million tons in 2020 (1), partially meeting the increasing global demand for food. By 2030, the production is estimated to increase by 15%. However, aquaculture also increases the global environmental burden, negatively impacting environments through eutrophication of effluent-receiving ecosystems and the widespread use of antibiotics (2–4). In response to this concern, the Food and Agriculture Organization of the United Nations (FAO) released the “Blue Transformation Roadmap 2022–2030” to achieve sustainable aquaculture systems (1). One task is to effectively manage the adverse impacts of aquaculture on global ecosystems.

Fish diseases cause significant economic losses and are among the biggest threats to aquaculture sustainability (5–8). Disease prevention is crucial for achieving sustainable management by minimizing the unproductive input of nutrients into the environment and reducing antibiotic dosage. An important step toward this goal is monitoring fish health and detecting diseases early to reduce spread, especially for diseases without commercially available vaccines. One such disease is bacterial cold-water disease (BCWD) (9), which is caused by *Flavobacterium psychrophilum* (10). *F. psychrophilum* infects ayu (*Plecoglossus altivelis*), especially in Asian countries (11), as well as salmonids such as rainbow trout (*Oncorhynchus mykiss*) and Atlantic salmon (*Salmo salar*), and is thus a threat to aquaculture worldwide (12). BCWD is first recognized through visible symptoms or death, and subsequently diagnosed by sampling tissues and culturing of *F. psychrophilum* or specific PCR targeting *F. psychrophilum* (12, 13), often leading to delayed treatment. Thus, identifying indicators that allow early detection of fish health status would be useful for enabling prompt intervention.

Gut microorganisms play an important role in the health and disease status of vertebrates, including mammals and fish. Microbiome analyses have revealed changes in the microbial community composition in the intestines of infected fish, serving as biological markers for disease (14–18). Recent advances in metabolomic analyses have also identified several potential chemical markers in pathogen-infected fish (19–23). However, these studies have the disadvantage of being invasive owing to the use of tissue or plasma for analysis, and the results reflect only the status of the individual examined, not the entire fish population in the culture tank.

In this study, we focused on examining fish feces accumulated at the bottom of rearing tanks as a source of non-invasive diagnostic biomarkers of bacterial infection. Feces in the tank can be collected non-invasively, providing a holistic view of the overall health of the fish population. The microbial community profiles and metabolites in fish feces are known to change in response to feeding type (24) and exposure to phenanthrene (25). Recently, Sun et al. (26) described changes in the fecal metabolome of zebrafish at different ages and after exposure to perfluorobutanesulfonate. However, to our knowledge, no study has evaluated whether microorganisms or their metabolites in fish feces can serve as indicators of bacterial infection. To this end, we employed multi-omics analyses, including metagenomics (16S rRNA gene amplicon sequencing and shotgun metagenomics) and metabolomics using nuclear magnetic resonance (NMR), to elucidate the microbiological and metabolic changes in the feces of *P. altivelis* infected with *F. psychrophilum*. Additionally, we measured cortisol levels in the feces as a generally accepted stress marker in fish (27–30). Finally, fish tissues were used for 16S rRNA gene amplicon sequencing and cortisol measurements to determine how well the results of the feces analyses reflected the conditions in the fish body.

## RESULTS

### Infection challenge test

**Fig 1:**
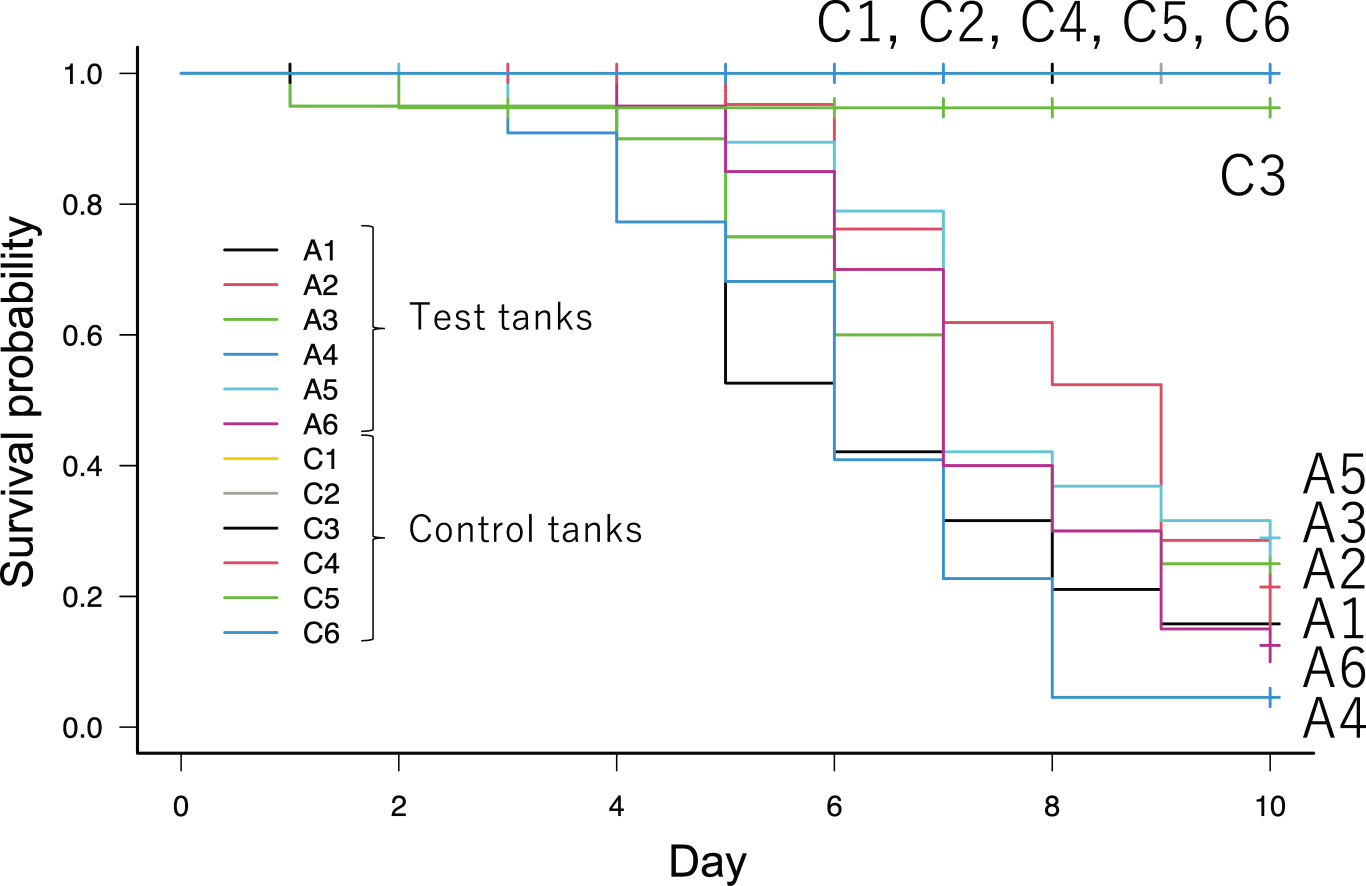
Kaplan-Meier graph depicting survival probability of *Plecoglossus altivelis* after exposure to *Flavobacterium psychrophilum* (test tanks A1–A6) and that of non-infected *P. altivelis* (control tanks C1–C6).

The test fish began to die 3 days after the infection challenge (Fig. 1). Most of the dead fish exhibited typical symptoms of BCWD, such as skin and jaw erosion (Fig. S1). Mortalities in the test tanks on day 10 were 63.2%–90.0%, whereas those in the control tanks were 0%–5.3%. The difference in mortality rates between the control and test tanks was statistically significant, as determined by the log-rank test (*P* < 0.05). *F. psychrophilum* was detected following culturing of the spleen or kidney of the dead fish from all of the test tanks. These results indicated that the infection challenge test using *F. psychrophilum* was successfully conducted. Fish in the test tank began to exhibit anorexia after day 4; thus, the amount of feed was adjusted to reduce leftovers (Table S1).

### Water quality

During the experiment, ammonium concentrations in the rearing water ranged from 0.1 to 2.5 mg N L^−1^ (Table S2A). Nitrate and nitrite concentrations were below 0.4 and 0.1 mg N L^−1^, respectively (Table S2B and C).

### Microbiome analysis using 16S rRNA gene amplicon sequencing

The number of non-chimeric sequence reads used for the microbiome analysis ranged from 23,112 to 119,209. The rarefaction curve indicated that sequence depths were sufficient to represent the bacterial community (Fig. S2). The total number of features was 4,968. Three alpha diversity metrics (chao1, Faith's phylogenetic diversity [Faith PD], and observed features) showed that the microbial communities in the intestines of dead fish were of lower diversity than those in the intestines of control fish (Fig. S3). Microbial communities in the intestines of surviving fish in the test tanks at the end of the experiment exhibited intermediate diversity between those in the intestines of dead and control fish, although a significant difference was observed only when comparing with that of control fish (Fig. S3). Shannon index of the water from the test tanks showed significantly higher diversity values than the water from the control tanks (Fig. S3). There was no significant difference in the feces alpha diversity between the test and control tanks, both before and after infection (Fig. S3).

**Fig 2:**
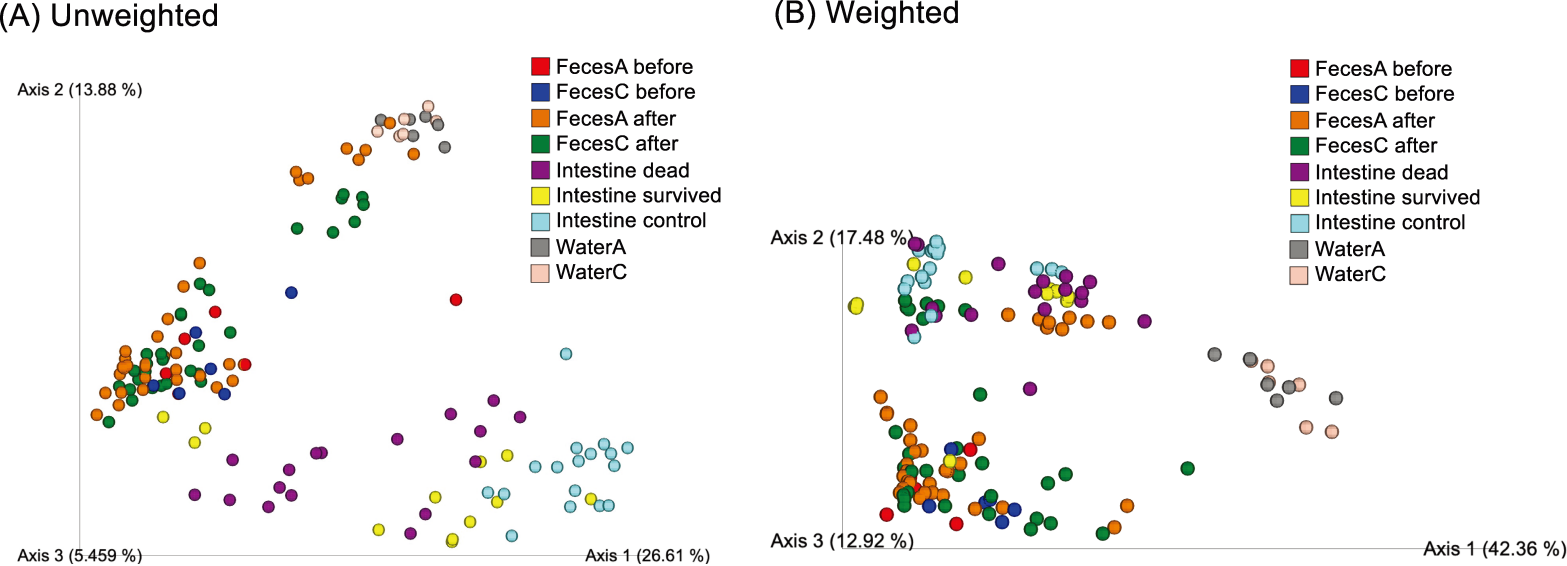
Principle coordinates analysis plots of microbial communities in feces before and after the infection challenge, intestines (dead, survived, and control), water, and feed. Data are presented according to (**A**) unweighted and (**B**) weighted UniFrac.

**Fig 3:**
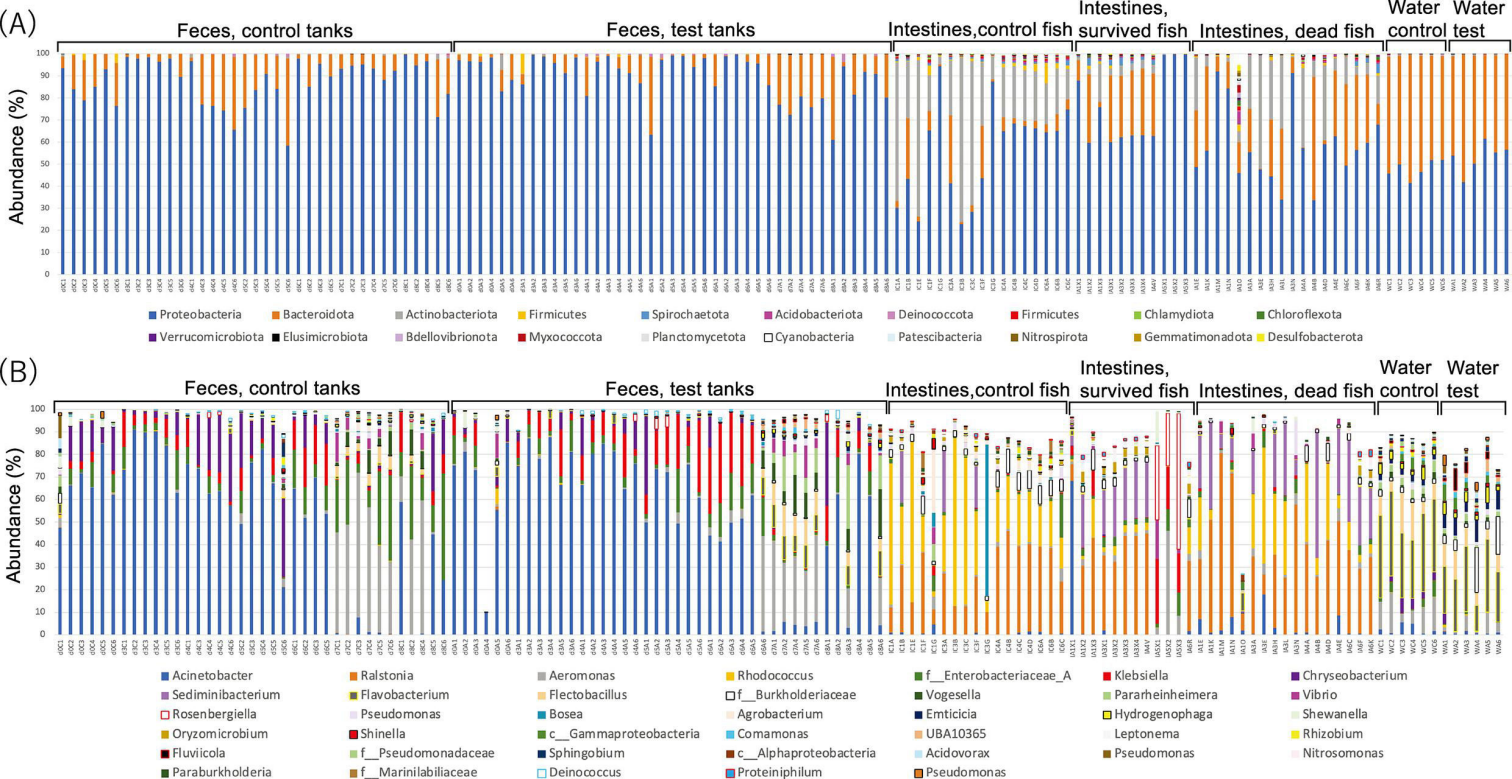
Relative microbial compositions in feces, intestines, and water at the (**A**) phylum (top 20 are shown) and (**B**) genus (top 40 are shown) levels.

Principal coordinate analysis (PCoA) based on both weighted and unweighted Unifrac distances showed distinct clustering of microbial communities in the intestines of dead fish and control fish (*q* < 0.01; Fig. 2A and B). Microbial communities in the intestines of surviving fish also differed from those of dead and control fish based on both weighted and unweighted Unifrac distances (*q* < 0.05; Fig. 2A and B). Members of the phyla Proteobacteria, Actinobacteria, Bacteroidota, and Firmicutes were the dominant microbial populations in the intestine (Fig. 3A). At the genus level, *Ralstonia*, *Rhodococcus*, *Sediminibacterium*, and an unknown group in the family Burkholderiaceae (hereafter, Burkholderiaceae group) were dominant in the intestines (Fig. 3B). Microbial communities in the feces were distantly related to those in the intestines and water based on both weighted and unweighted Unifrac distances (*q* < 0.01; Fig. 2A and B). PCoA revealed no difference between the microbial communities in the feces from the test and control tanks before infection. After infection, unweighted Unifrac-based analysis detected a difference in feces between the test and control tanks (*q* < 0.01). When microbial communities in the feces were compared each day, the differences between the test and control tanks were significant on days 3, 4, 5, and 7 (*q* < 0.01) based on unweighted Unifrac analysis, whereas weighted Unifrac analysis revealed a difference only on day 7 (*q* < 0.01; Fig. S4A and B). Proteobacteria, Bacteroidota, and Firmicutes dominated the fecal microbial community at the phylum level (Fig. 3A). At the genus level, *Acinetobacter*, *Aeromonas*, *Klebsiella*, and *Chryseobacterium* were dominant (Fig. 3B). The microbial communities in the water of the test tanks were significantly different from those in the control tanks (*q* < 0.05). At the phylum level, Proteobacteria and Bacteroidota were the major microbial constituents in the water samples (Fig. 3A). At the genus level, *Flavobacterium*, among which *F. psychrophilum* constituted <1% both in test and control tanks, and *Flectobacillus* dominated in the water samples (Fig. 3B).

### Microbial component features in the intestines of dead, control, and surviving fish

Analysis of composition of microbiomes (ANCOM) was used to detect genera with differential abundances between dead and control fish. *Oryzomicrobium*, *Leptonema*, *Nitrosomonas*, *Agriterribacter*, an unknown group in the order Bacteroidales, *Dokdonella*, *Proteiniphilum*, and *Thiobacillus* were detected (Table S3A). These genera were more abundant in control fish than in dead fish. Among these eight genera, ANCOM revealed that seven, excluding *Dokdonella*, were also more abundant in surviving fish than in dead fish (Table S3A). In addition to the aforementioned eight genera, Welch’s *t*-test revealed that among the genera with a >1% abundance, the relative abundances of *Rhodococcus*, Burkholderiaceae group, and an unknown group in the family Marinilabiliaceae were lower, and the abundance of *Sediminibacterium* was higher in dead fish than in control fish (Table S3A). Relative abundances of these genera in surviving fish showed similar trends as observed for dead fish (Table S3A). *Sediminibacterium* could be an intestinal biomarker of infection. *F. psychrophilum* was detected at an average of 0.6%, 0.2%, and 0.002% in dead, surviving, and control fish, respectively.

### Microbial component features of feces in test tanks

ANCOM of all of the feces data after the challenge found a difference in the abundance of *Agrobacterium*, *Cypionkella*, *Sphingopyxis*, and *Rhizobium* between the test and control tanks (Table S3B). Welch’s *t*-test also found that among genera with >0.5% abundance on average, *Flavobacterium*, *Pararheinheimera*, *Fluviicola*, *Comamonas*, *Cypionkella*, *Shinella*, and *Klebsiella* were more abundant in test tanks than in control tanks, while *Agrobacterium*, *Chryseobacterium*, and an unknown group of Enterobacteriaceae were less abundant (Table S3B). *F. psychrophilum* was significantly more abundant in feces from test tanks (0.45% on average) than from control tanks (0.00% on average).

### Microbial components in feces associated with mortality

**Fig 4:**
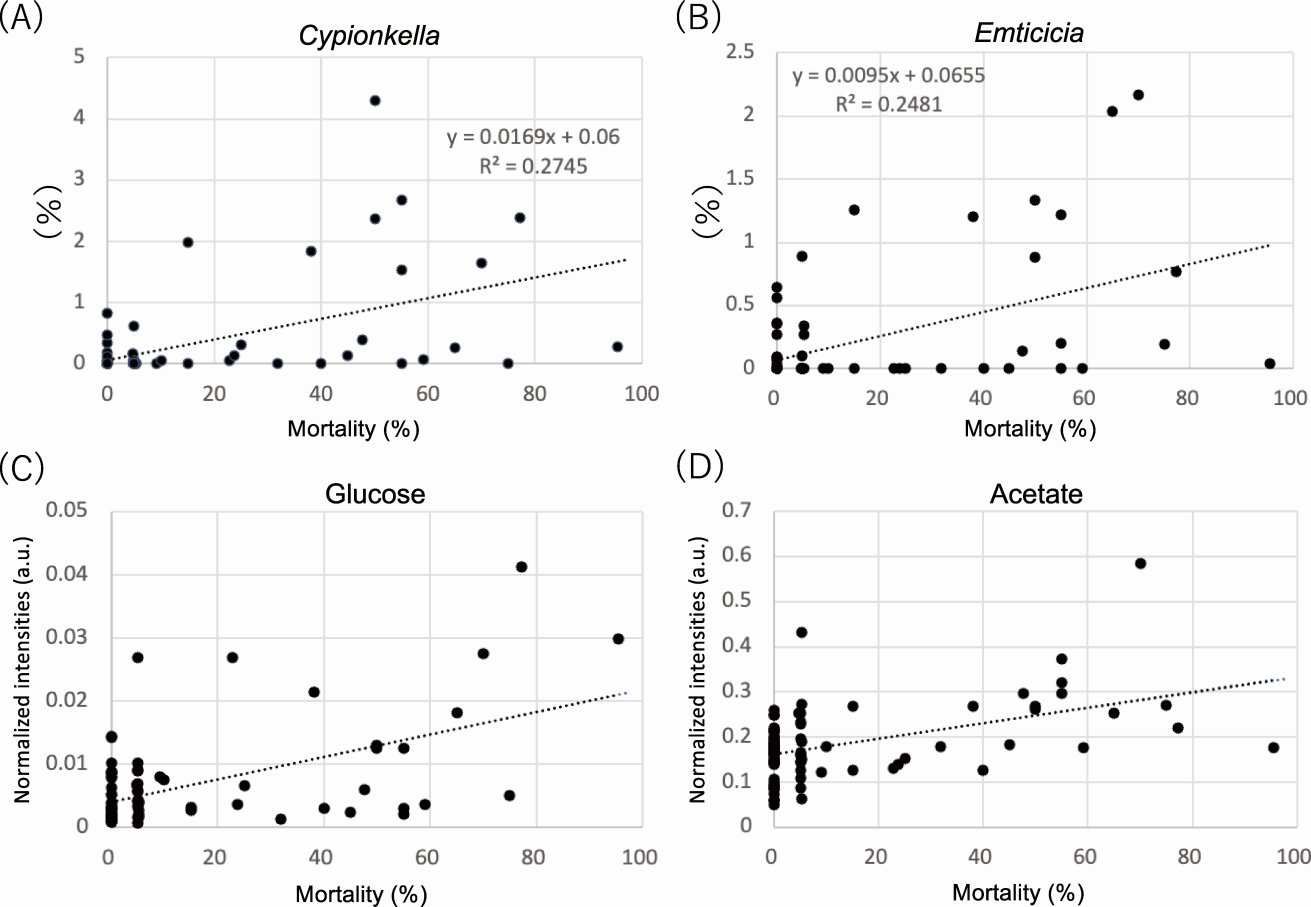
Correlations between mortality and the relative percentages of (**A**) *Cypionkella* and (**B**) *Emticicia* and the levels of (**C**) glucose and (**D**) acetate.

The impact of the microbial components on mortality was examined using stepwise regression analysis. The relative abundances of *F. psychrophilum* and the 12 genera specified earlier were used as explanatory variables. The relative abundance of *Cypionkella* was most positively correlated with mortality (*R*^2^ = 0.27; Fig. 4A), followed by that of *Emticicia* (*R*^2^ = 0.25; Fig. 4B). The pathogen *F. psychrophilum* showed a weak correlation with mortality (Fig. S5A).

**Fig 5:**
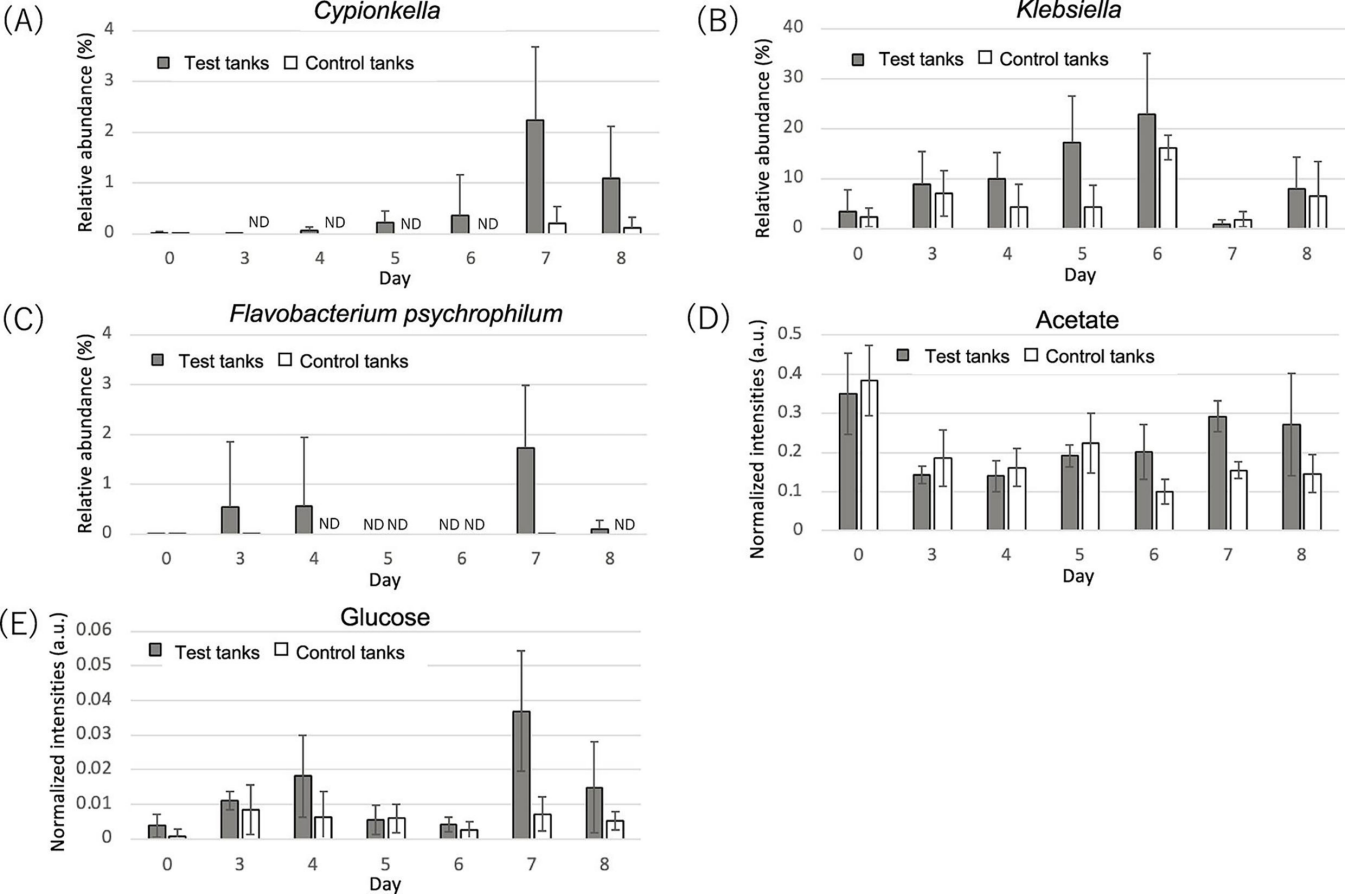
Changes in the relative abundance of (**A**) *Cypionkella*, (**B**) *Klebsiella,* and (**C**) *Flavobacterium psychrophilum,* and levels of (**D**) acetate and (**E**) glucose in feces obtained from test and control tanks. ND, not detected. Statistical bars show standard deviation.

Based on our findings, *Cypionkella,* whose abundance correlated with mortality, and *Klebsiella* and *F. psychrophilum*, which were abundant in the feces of test tanks, are considered as possible biomarkers. Relative abundances of these groups began to increase after infection and peaked on day 6 or 7 (Fig. 5A to C). Specifically, a significant difference in the relative abundance of *Cypionkella* was observed on day 4 between the test and control tanks.

### Metabolome analysis

From the 143 metabolic signals in the region of interest (ROI), major metabolites, such as amino acids, organic acids, sugars, and amines, were annotated using two-dimensional NMR and the metabolite database. Based on the normalized annotated metabolites observed in the feces through NMR analysis (Table S4), the levels of acetate, glucose, and formate were significantly different between the control and test tanks. Glucose and acetate levels were significantly higher in the feces from test tanks (2.4 and 1.3 times, *P* < 0.05 and 0.01, respectively), while formate levels were lower (0.2 times, *P* < 0.001). A significant increase in acetate and glucose levels was observed on days 6 and 7, respectively (Fig. 5D and E).

### Metabolites associated with mortality

The impact of the important metabolites in the feces (acetate, glucose, formate, and cortisol) on mortality was examined using stepwise regression analysis. Glucose and acetate levels were significantly (*P* < 0.05) and positively correlated with mortality (*R*^2^ = 0.2982 and 0.2249, respectively; Fig. 4C and D). In contrast, cortisol and formate levels showed a weak correlation with mortality (*R*^2^ = 0.03 and 0.0457, respectively; Fig. S5B and C).

### Microbial components associated with the metabolome changes in the feces

Spearman’s rank correlation coefficients were calculated to assess the correlation between metabolites (acetate, glucose, and formate) and the top 45 bacterial genera in the feces (Fig. S6). The level of acetate was positively correlated with *Deefgea* (ρ = 0.42, *P* < 0.001) and *Cypionkella* (ρ = 0.38, *P* < 0.001). Glucose levels were positively correlated with *Shinella* (ρ = 0.38, *P* < 0.001) and *Deefgea* (ρ = 0.37, *P* < 0.001). The level of formate was positively correlated with *Aliivibrio* (ρ = 0.44, *P* < 0.001).

### Genomic characterization of microorganisms

Following metagenomic shotgun sequence analysis of nine feces samples, we obtained 77 high-quality (50.34%–99.94% completeness and 0%–36.62% contamination, except for two pan-genomes) assembled bins (Table S5). Acetate emerged as an indicator of infection in feces, showing the strongest correlation with *Deefgea* and *Cypionkella*. We successfully recovered the draft genome of *Cypionkella* (AY.29). This metagenomic bin contained genes involved in the utilization of various sugars, such as glucose, fructose (AY.29_01170), and mannose (AY.29_02687, AY.29_02096). It also contained genes involved in the production of lactate (AY.29_02180) and acetate (AY.29_00625, AY.29_02417) (Table S5). We could not obtain the *Deefgea* bin, but a partial 16S rRNA gene sequence of the major amplicon sequence variant (ASV) in *Deefgea* was 100% identical to that of *Deefgea piscis* strain D17 isolated from Korean fish (31). The genome of *D. piscis* (T06622) was also found to contain an acetate kinase gene (HQN60_03035).

**Fig 6:**
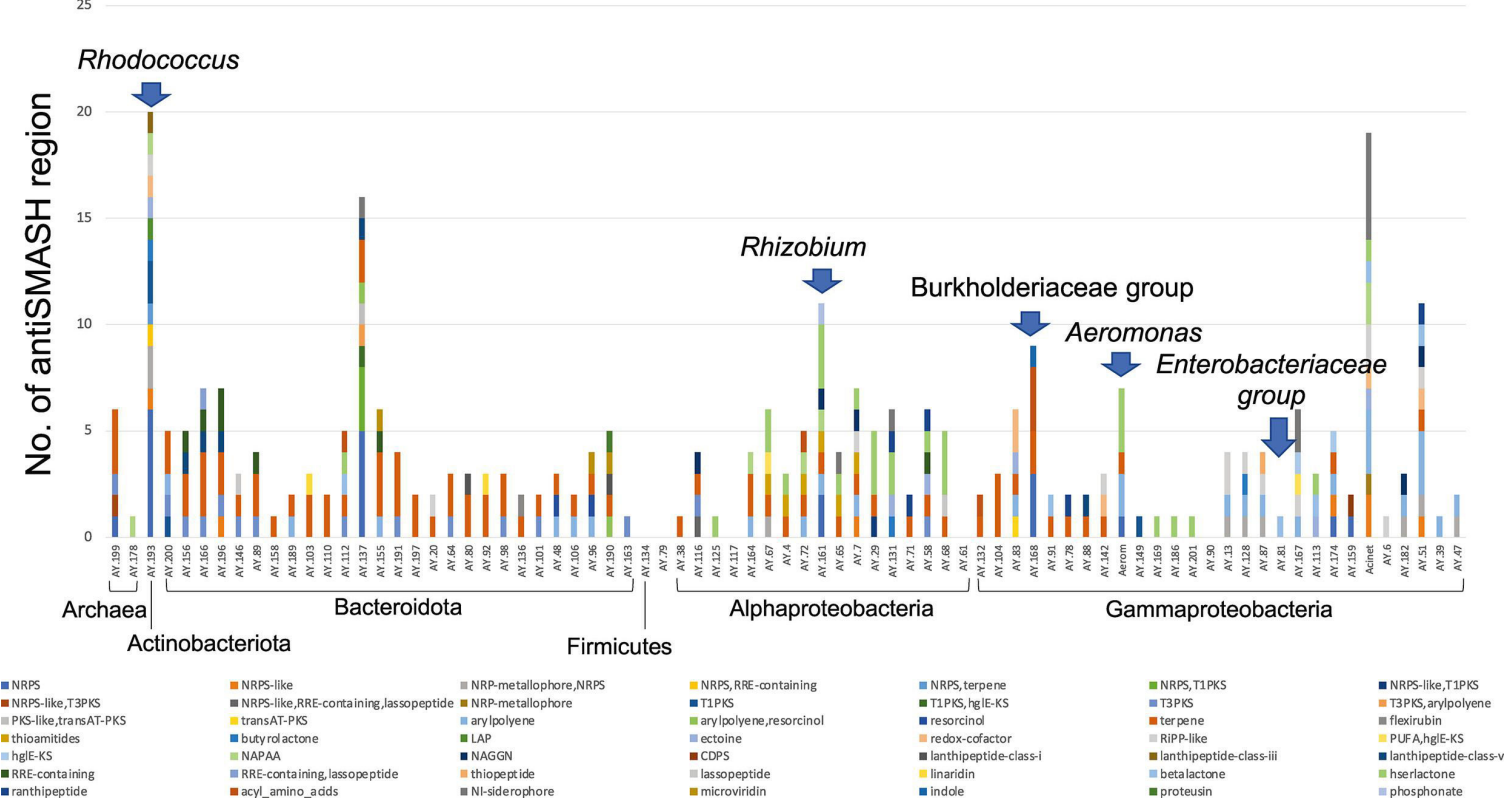
Number of antiSMASH regions detected in bins obtained in this study.

Among the genera with a relatively high abundance in the intestines of the control fish, we successfully recovered a *Rhodococcus* bin (AY.193). The partial 16S rRNA gene sequence of the major ASV in the Burkholderiaceae group was 100% identical to that of *Pelomonas puraquae* (AM501439), and a *Pelomonas*-related bin (AY.168) was also recovered. Additionally, we retrieved bins of microorganisms that were more abundant in feces from control tanks than those from test tanks, including the Enterobacteriaceae group (AY.81), the sequence of which was 100% identical to that of *Plesiomonas shigelloides* (X60418) and *Rhizobium* (AY.161). We performed antiSMASH analysis to search for secondary metabolite biosynthesis gene clusters. Analysis of bins AY.193, AY.168, and AY.161 showed that the microbes in intestines or feces of healthy fish had a higher number of antiSMASH regions (Fig. 6). In particular, AY.193 of *Rhodococcus* contained the highest number of antiSMASH regions, i.e., 20, including 13 non-ribosomal peptide synthetases and polyketide synthase coding regions.

### Cortisol

**Fig 7:**
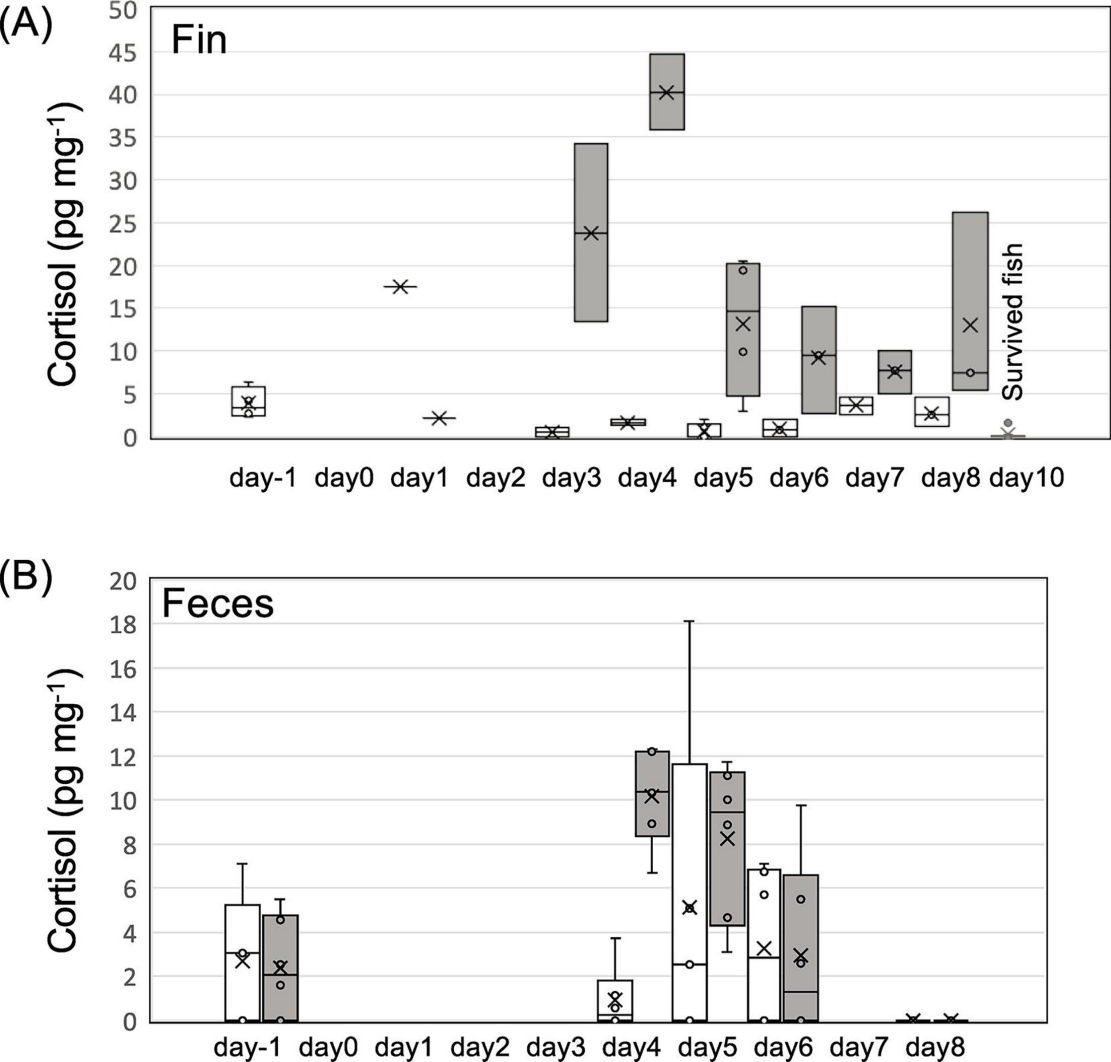
Cortisol concentrations in (**A**) fins and (**B**) feces of *P. altivelis*. Gray boxes represent test tanks and white boxes represent control tanks. Day 10 data in panel **A** represent surviving fish from the test tanks.

Cortisol concentrations in the caudal fin of fish before the infection challenge were 3.9 ± 1.9 pg mg^−1^ wet weight (Fig. 7A). For the fish in the control tanks, cortisol concentrations remained low (2.4 ± 3.8 pg mg^−1^), with one exception on day 1. Cortisol concentrations in dead fish were 15.1 ± 12.6 pg mg^−1^, peaking on day 4, and significantly higher than those in control fish after the infection challenge (*P* < 0.001; Fig. 7A). In surviving fish, cortisol concentrations were 0.2 ± 0.5 pg mg^−1^. In feces, cortisol concentrations were 2.5 ± 2.5 pg mg^−1^ dry weight before the infection challenge (Fig. 7B). After the infection challenge, cortisol concentrations were significantly elevated in feces from test tanks (7.1 ± 4.5 pg mg^−1^) compared with that in feces from control tanks (3.0 ± 4.7 pg mg^−1^, *P* < 0.05), and they also peaked on day 4.

## DISCUSSION

In this study, we showed that the levels of several potential biomarkers in fish feces collected from aquaculture tanks were significantly altered by bacterial infection and could be used to detect BCWD in fish.

### Glucose

We found a positive correlation between mortality and glucose levels in feces; glucose is known as a stress marker in fish (32–34). Robertson et al. (33) reported that plasma glucose levels increased after reducing salinity in red drum *Sciaenops ocellatus* infected by an unidentified pathogen. Therefore, the increase in fecal glucose in the current study might reflect stress levels in fish. However, several reports on infected fish contradict this hypothesis. In the livers of dying tilapia infected with *Edwardsiella tarda*, a decrease in glucose levels was noted (22). A similar decrease in serum glucose levels was also observed in emaciated *Takifugu rubripes* caused by myxosporean emaciation disease (35). In mammals infected with pathogenic bacteria, anorexia plays an important role in reducing glucose levels and enhancing ketogenesis, which is necessary for limiting the reactive oxygen species induced by antibacterial inflammation (36). It is speculated that the diseased fish not only decreased feed intake, but may have also decreased absorption of glucose in the intestine, leading to increased glucose levels in the feces.

### Acetate

We found that acetate in feces could be a biomarker of BCWD, and *Deefgea* and *Cypionkella*, which possess the acetate kinase gene, may be involved in acetate production. The observed increase in glucose concentrations may have enhanced the metabolic activities of these microorganisms producing acetate in the feces. In humans, short-chain fatty acids (SCFAs) are recognized as key metabolites produced by gut microbes, affecting health and disease (37). Thus, SCFAs in the intestine may also play a crucial role in fish health and disease. An interesting future research direction would be to analyze the SCFA profiles in the intestines and feces of fish and determine their relevance to health and disease.

### Distinct microbial communities in feces, intestine, and water

We observed significant differences in the microbial communities between the feces, intestine, and water. Microbial structure is known to differ between intestinal mucosa and its contents (18). Our use of the whole intestine, including mucosa and contents, for analysis may have contributed to the inconsistency in results between feces and intestine. In this study, fish were fed in the evening and feces were collected the following morning. This period in the water may have caused some changes in the microbial community. Rinsing of feces is reported to be effective in revealing the true nature of metabolites packed inside the feces of fish (38). Changes in the microbiome in feces over time after extrusion into water and the effect of rinsing should be further examined in the future.

The microbial community compositions in the intestine and feces were distinct from those observed in the water. This is in line with previous studies on other fish (39, 40) showing that fish intestines harbor distinct microbial communities. While we observed a significant difference between fecal and water microbial communities on day 7, some genera, such as *Aeromonas*, *Flectobacillus*, and *Pararheinheimera*, were commonly found between the two environments. This suggested there was a link between the microbes in the feces and water environment. *Flectobacillus roseus* is a free-living bacteria (41). We speculated that microorganisms originating from the fish and capable of free-living, such as *F. roseus,* proliferated in the surrounding water.

### Microbial biomarkers in feces and intestine

*F. psychrophilum* shows promise as a specific biomarker for BCWD. Generally, bacterial tests involving invasive sampling of tissues, such as the kidney or spleen, are used for diagnosis. On average, the relative percentage of *F. psychrophilum* in the microbiome was 0.6% in the intestines of dead fish, 0.5% in feces after infection challenge, and 0.2% in the water of test tanks. This shows that the feces contained a relative abundance of *F. psychrophilum* comparable to that of the intestine, supporting the monitoring of *F. psychrophilum* for BCWD detection.

Among other microorganisms, *Cypionkella* was related to both acetate production and mortality, making it another candidate biomarker. However, it is not clear why *Cypionkella* proliferated in the feces of infected fish. *Klebsiella* was the most dominant bacterial genus enriched in feces from the test tanks compared to that in the control tanks. The *Klebsiella* bin (AY.87, *Raoultella terrigena*) also contained a gene encoding acetate kinase, suggesting its involvement in acetate production as well as that of *Cypionkella. Klebsiella* is a group of opportunistic pathogens found not only in humans, but also in fish (42, 43). Generally, opportunistic pathogens proliferate when the host is under stress (44). A significant increase in opportunistic bacteria has been reported in the skin of diseased fish (45, 46). Proliferation of *Klebsiella* in the feces of infected fish may also be associated with stress in ayu as indicated by the increase in cortisol concentrations.

The intestinal microbiome of the infected fish also exhibited specific features. The decrease in alpha diversity indices in infected fish was consistent with previous reports on other diseased fish, such as those with furunculosis caused by *Aeromonas salmonicida* (14) and *Vibrio anguillarum* (15). Furthermore, the abundance of potentially beneficial microbes with many secondary metabolite biosynthetic genes in their genome decreased in infected fish as well as in feces. This suggests that microorganisms indigenous to healthy fish play an important role in preventing infection by pathogens, and that dysbiosis occurs in fish infected with *F. psychrophilum*. The microbiome of the surviving fish showed intermediate characteristics between that of dead and control fish. This may explain why the differences in feces between the test and control tanks, the former of which contained both moribund and more healthy fish, were less clear than those of the intestines when comparing dead and control fish.

### Cortisol

Generally, cortisol in fish is measured using invasive sampling, including plasma (47). Cao et al. (48) and Webster et al. (30) showed that cortisol concentrations in fish feces squeezed out directly from the anus correlated well with plasma cortisol levels. In the current study, the concentration of cortisol in the fins increased after the infection challenge; this was also found in the feces (Fig. 7). Thus, cortisol levels in tank feces also reflected the average stress conditions of the fish. When released into the water, the half-life of cortisol is 16 h at 12°C (49), suggesting that frequent collection of feces is preferable. Previous reports on the analysis of cortisol in fish feces have used various extraction methods, including organic solvents such as ethanol (30), dichloromethane (50), and methanol, and also phosphate-buffered saline (PBS), which is effective for gills and mucus (47). Our preliminary results showed that PBS extraction of feces yielded higher values of cortisol than did methanol extraction (data not shown). Here, we showed that cortisol in feces can also be measured after PBS extraction, which saves time and costs compared with those of solvent extraction. Elevated cortisol levels in fish are associated with ammonium concentration, handling (51), rearing density (52), transport (32), suspended sediments (53), and bacterial infections (33). In the current study, most of the environmental conditions were identical between the test and control tanks. Thus, the elevated cortisol levels in the fins and feces were likely due to bacterial infection. Cortisol in feces is expected to be an indicator of a wide range of bacterial infections, including BCWD.

It is noteworthy that the peak in cortisol concentration in the feces was observed on day 4, earlier than that of the other possible biomarkers. However, cortisol concentrations in the feces decreased thereafter, while other candidate biomarkers peaked on day 6 or 7. This suggests that multiple biomarkers can complement each other to non-invasively detect changes in the health status of fish.

### Conclusion

In this study, we demonstrated that the abundance of microbes and the concentration of several metabolites in fish feces could potentially serve as useful biomarkers for the detection of BCWD in fish production systems. Health monitoring through feces may be a novel non-invasive diagnostic technique for a wide range of diseases, especially for inland aquaculture systems.

## MATERIALS AND METHODS

### Bacterial strain

*F. psychrophilum* strain KU190628-79, originally isolated from *P. altivelis,* was grown in FLP medium (54) at 16°C for 2 days until the optical density (OD) of the culture reached 0.3. It was then used for the infection challenge test. The number of colony forming units (CFU) mL^−1^ of strain KU190628-79 was determined using the drop-plate method (55).

### Infection challenge test

**Fig 8:**
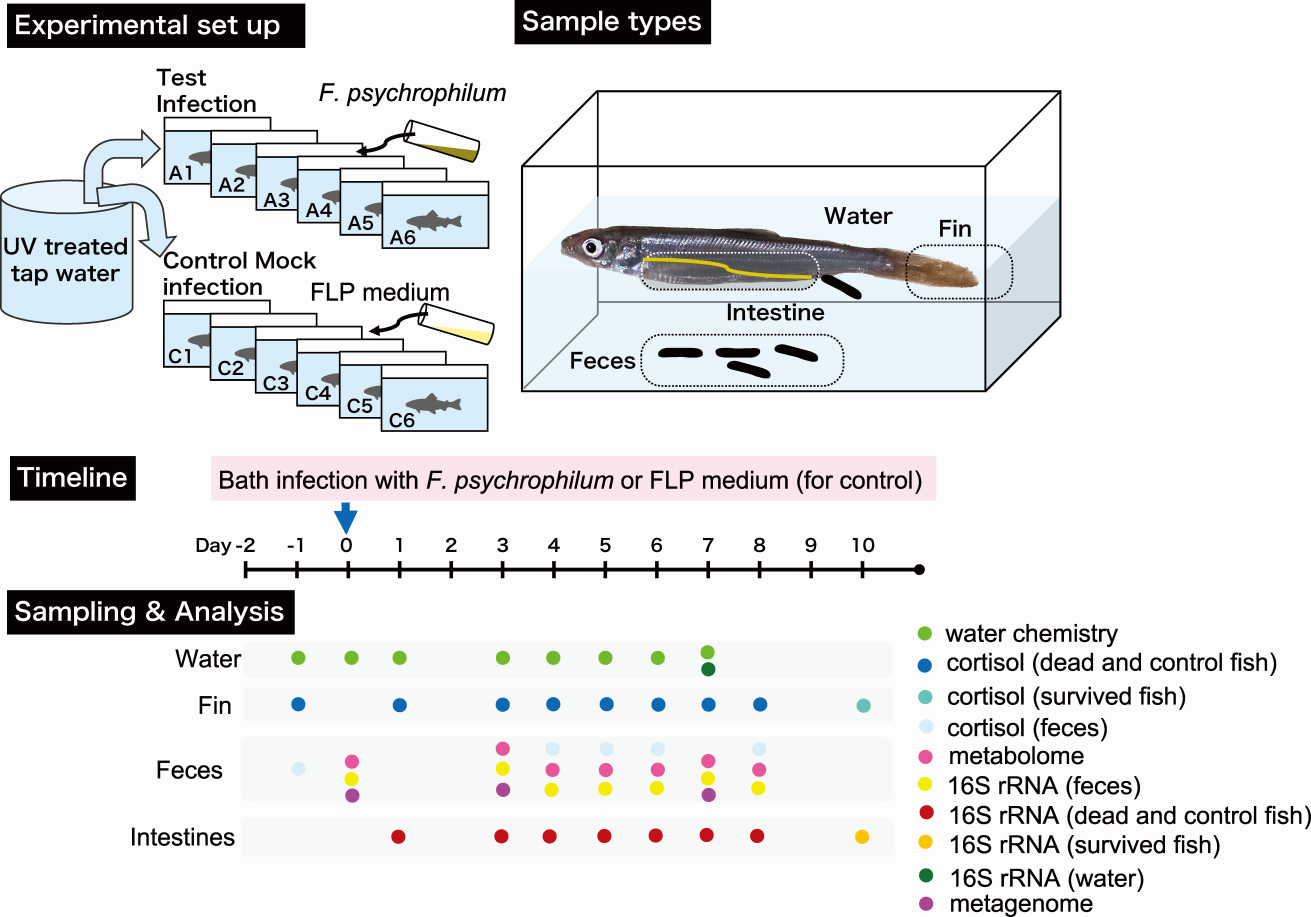
Experimental overview and sampling strategy.

The experimental overview and sampling strategy are presented in Fig. 8. *P. altivelis* larvae (average weight, 1.0 g) were purchased from Marinetech Co., Ltd. (Aichi, Japan). Three days pre-infection challenge, fish were brought into water containing 0.5% NaCl. Dechlorinated tap water was treated with activated carbon and UV light and maintained at 18°C in the storage tank before being introduced into 12 fish tanks. The fish were divided among the 12 tanks and initially acclimated in 10 L of dechlorinated tap water containing 0.5% NaCl, which was aerated using air stones without flowthrough. On the next day (designated as day −2), the water was replaced with fresh water (treated tap water), flowthrough was initiated at a rate of 0.3 mL s^−1^, and the fish were acclimated for an additional 2 days before the challenge test.

All fish were fed a commercial diet (Otohime C-2, Marubeni Nisshin Feed Co., Ltd, Tokyo, Japan), except on days 0 and 1, at an amount less than 3% of their total weight per day (Table S1). Leftover feed was removed by pipetting to avoid mixing with the feces. Six tanks were assigned as control tanks (C1–C6) and six others were assigned as infection challenge test tanks (A1–A6). There were 18–22 fish per tank on day 0. Water flowthrough was stopped on the day of infection (day 0), and then the fish were infected with *F. psychrophilum* by adding 10 mL of the culture at a final concentration of 1 × 10^6^ CFU mL^−1^. For the control tanks, 10 mL of FLP medium was added. After 24 h, the water was reduced to 20% vol and replenished with fresh water. This process was repeated three times before resuming the water flowthrough. The temperature in the tank was kept at 16.5°C–18.5°C during the experiment.

Daily mortalities were recorded, and infection by *F. psychrophilum* was confirmed for dead fish through culturing of the spleen and kidney on FLP agar at 18°C for 3–4 days. Yellow colonies were identified using matrix-assisted laser desorption/ionization-time of flight mass spectrometry.

### Feces and fish samples

Feces were collected from the bottom of the tank in the morning using a pipette. We could not collect samples on days 1 and 2 because feeding of the fish was withheld during exposure to *F. psychrophilum* (days 0 and 1) to prevent deterioration of the water quality, which is crucial for *P. altivelis* survival. After discarding excess water, the fecal samples were centrifuged at 2,000 × *g* for 5 min. Thereafter, the water phase was completely removed and the samples were stored at −80°C. Subsequently, the feces samples were lyophilized and used in metagenomic, metabolomic, and cortisol analyses. Moribund fish and fish that had recently died (hereafter, dead fish) were collected using autoclaved nets. Dead fish found in the morning were counted, but excluded from further analysis. When dead fish was sampled, an equivalent number of fish were also collected from the control tank. At the end of the experiment, fish that survived in the test tanks were also collected. After washing with sterilized water, the fish were placed on ice and disinfected with 70% ethanol before removal of the caudal fin and subsequent dissection. The caudal fin and the whole intestine were placed in 1.5 mL plastic tubes, and stored at −80°C.

### Analysis of ammonium, nitrate, and nitrite in the rearing water

An aliquot (10 mL) of the rearing water in each tank was sampled in the morning and evening and filtered using a 0.2 µm filter. Concentrations of ammonium, nitrate, and nitrite in the rearing water were measured as previously described (56).

### 16S rRNA gene sequencing analysis

DNA was extracted from feces from each tank (*n* = 78, 1–30 mg, dry weight) and intestines from individual fish (*n* = 17, *n* = 17, *n* = 12, for dead, control, and surviving fish, respectively; 11 mg–68 mg wet weight) using a FastDNA Spin Kit for Soil (MP Biomedicals, Irvine, CA, USA). We used this kit because a larger amount of DNA could be extracted from feces using this kit than when using two feces-specific DNA extraction kits (ISOSPIN Fecal DNA and ISOFECAL for Beads Beating, NIPPON GENE Co., Ltd., Tokyo, Japan; data not shown). Owing to the small size of the intestines, whole intestines, including the contents, were used for DNA extraction. Microbes in the rearing water on day 7 (*n* = 12) were also analyzed for reference. Rearing water (100 mL) was centrifuged at 6,500 × *g* for 10 min. After removing most of the supernatant, the remnant was vortexed and then centrifuged at 9,000 × *g* for 3 min. The collected cells were used for DNA extraction. Details of PCR, sequencing, and bioinformatic analysis using QIIME2 (57) are described in the supplementary material.

### Shotgun metagenomic sequence analysis

Feces from control tank C6, test tank A2, and test tank A4, sampled on days 0, 3, and 7 (9 samples in total), were used for shotgun metagenome sequence analysis using DNA extracted as described earlier. Details of the sequencing and bioinformatic analysis and binning process are described in the supplementary material.

### Metabolome analysis

For NMR measurements, fish feces were processed as previously described, with some modifications (58). Briefly, 5 mg of lyophilized feces was extracted with 600 µL of KPi (D_2_O) at 65°C for 15 min and centrifuged at 14,000 rpm for 5 min. For NMR observations, two-dimensional *J*-resolved (pulse sequence of jresgpprqf) and ^1^H-^13^C heteronuclear single quantum coherence (pulse sequence of hsqcetgp) spectra were measured at 25°C using a Bruker DRU-700 spectrometer equipped with a ^1^H inverse triple-resonance cryogenically cooled probe with z-axis gradients (Bruker BioSpin GmbH, Rheinstetten, Germany), as previously described (58, 59). Peak definition as ROIs and subsequent metabolite annotation were performed using our standard database, InterSpin (60, 61).

### Cortisol measurement

Cortisol concentrations were measured in the caudal fins before the infection challenge (*n* = 4), as well as in the dead fish (*n* = 18), control fish (*n* = 20), surviving fish (*n* = 9), and feces samples (*n* = 56). PBS (200 µL) was added to a 1.5 mL plastic tube containing fin tissue (5.7 ± 1.8 mg), which was then subjected to homogenization. For feces (3.1 ± 1.9 mg), PBS (200 µL) was added and the sample was vortexed for 2 min. After centrifugation at 4,000 × *g* for 10 min, the supernatant was analyzed using a Cortisol ELISA Kit (Cayman Chemical, Ann Arbor, MI, USA) according to the manufacturer’s instructions.

### Statistical analyses

During the infection challenge test, survival analysis was conducted by plotting Kaplan-Meier curves, and statistical differences were assessed using a log-rank test. The significance of the differences in the beta-diversity metrics of the microbial community was assessed using the pairwise permutational multivariate analysis of variance (PERMANOVA) test and the “qiime diversity beta-group-significance” command (62). A false discovery rate-corrected *P*-value (*q* < 0.05) was considered as statistically significant. Statistical significance of the data obtained by chemical and microbiological analyses was evaluated using Welch’s *t*-test. Spearman’s rank correlation coefficient and multivariate analysis by stepwise regression were used to calculate correlations among variables using the R software package ver. 4.3.0 (63) in RStudio ver. 2023.03.1+446.

## Data Availability

The raw sequence and binned metagenome data generated in this study were deposited in the DDBJ Sequence Read Archive under BioProject ID PRJDB16637 (DRA017116 and DRA017212).

## SUPPLEMENTAL MATERIAL

Supplemental figures msphere.00301-24-s0001.pdf Fig. S1 to S6.

Supplemental text msphere.00301-24-s0002.docx Supplemental text for Materials and Methods.

Table S1 msphere.00301-24-s0003.xlsx Amount of feed for control and test tanks.

Table S2 msphere.00301-24-s0004.xlsx Ammonium, nitrate , and nitrite concentrations in the rearing water taken form control and test tanks.

Table S3 msphere.00301-24-s0005.xlsx Relative abundances of bacterial genera with differential abundance between intestine and feces.

Table S4 msphere.00301-24-s0006.xlsx Detected metabolites in the NMR spectra of feces samples.

Table S5 msphere.00301-24-s0007.xlsx Summary of the metagenomic bins from feces samples in this study.

Table S6 msphere.00301-24-s0008.xlsx Original bins and their phylogenetic positions before merging pangenomes.

ASM does not own the copyrights to Supplemental Material that may be linked to, or accessed through, an article. The authors have granted ASM a non-exclusive, world-wide license to publish the Supplemental Material files. Please contact the corresponding author directly for reuse.
