# Supplementary material for: Fecal metagenomic and metabolomic analyses reveal non-invasive biomarkers of Flavobacterium psychrophilum infection in ayu (Plecoglossus altivelis)
Source: mSphere. 2024 Jun 17;9(7):e00301-24. doi: 10.1128/msphere.00301-24 (PMC11288038; doi:10.1128/msphere.00301-24)
Supplement: Supplemental figures — Fig. S1 to S6. [file msphere.00301-24-s0001.pdf]

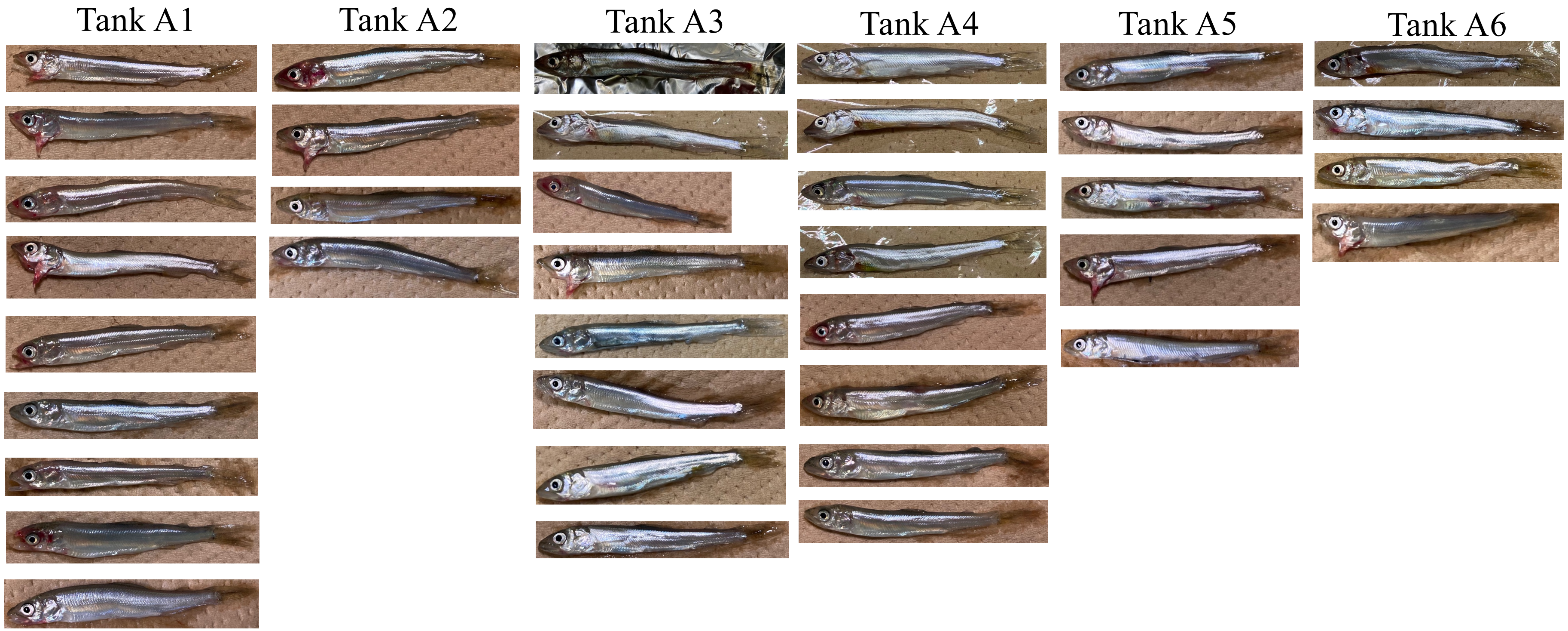

Figure S1. Symptoms of dead fish from test tanks.

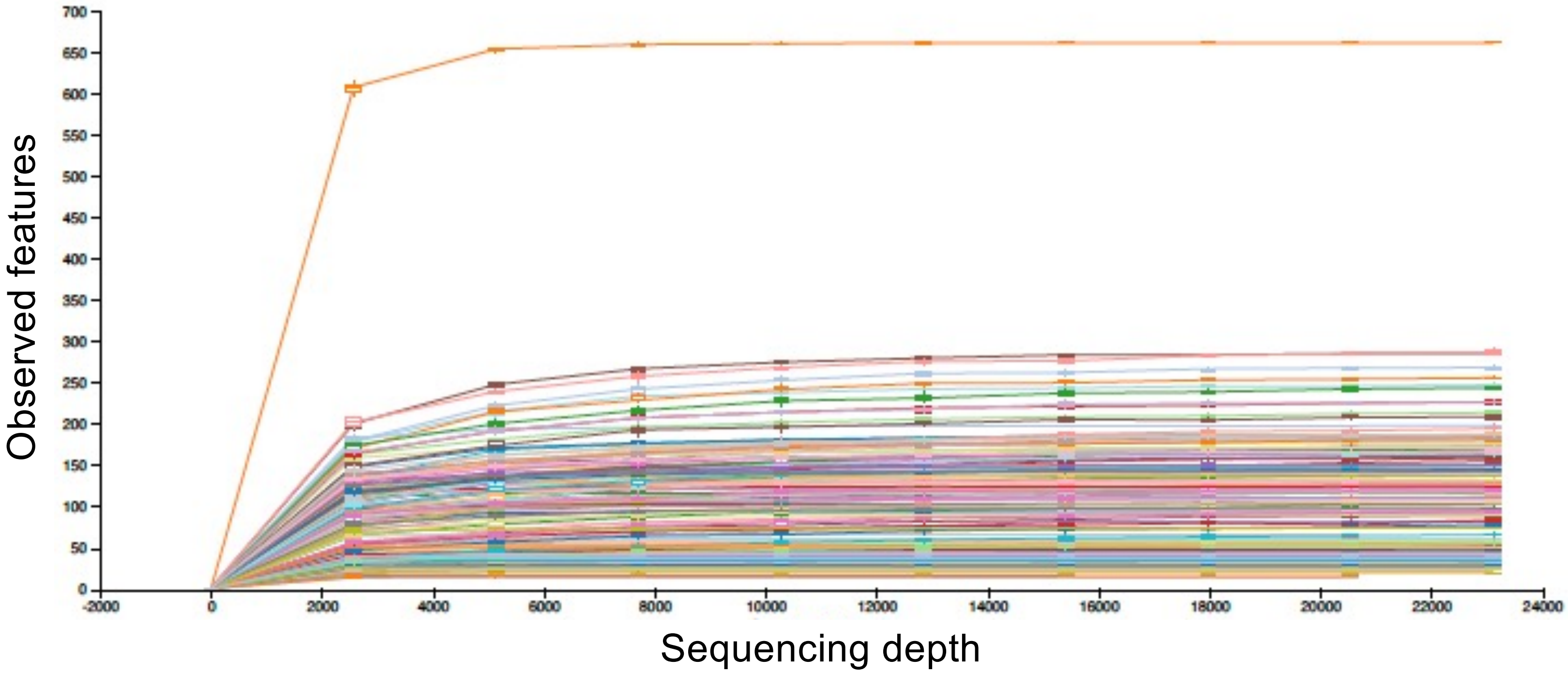

Figure S2. Rarefaction curves showing observed features in feces, intestines, and water.

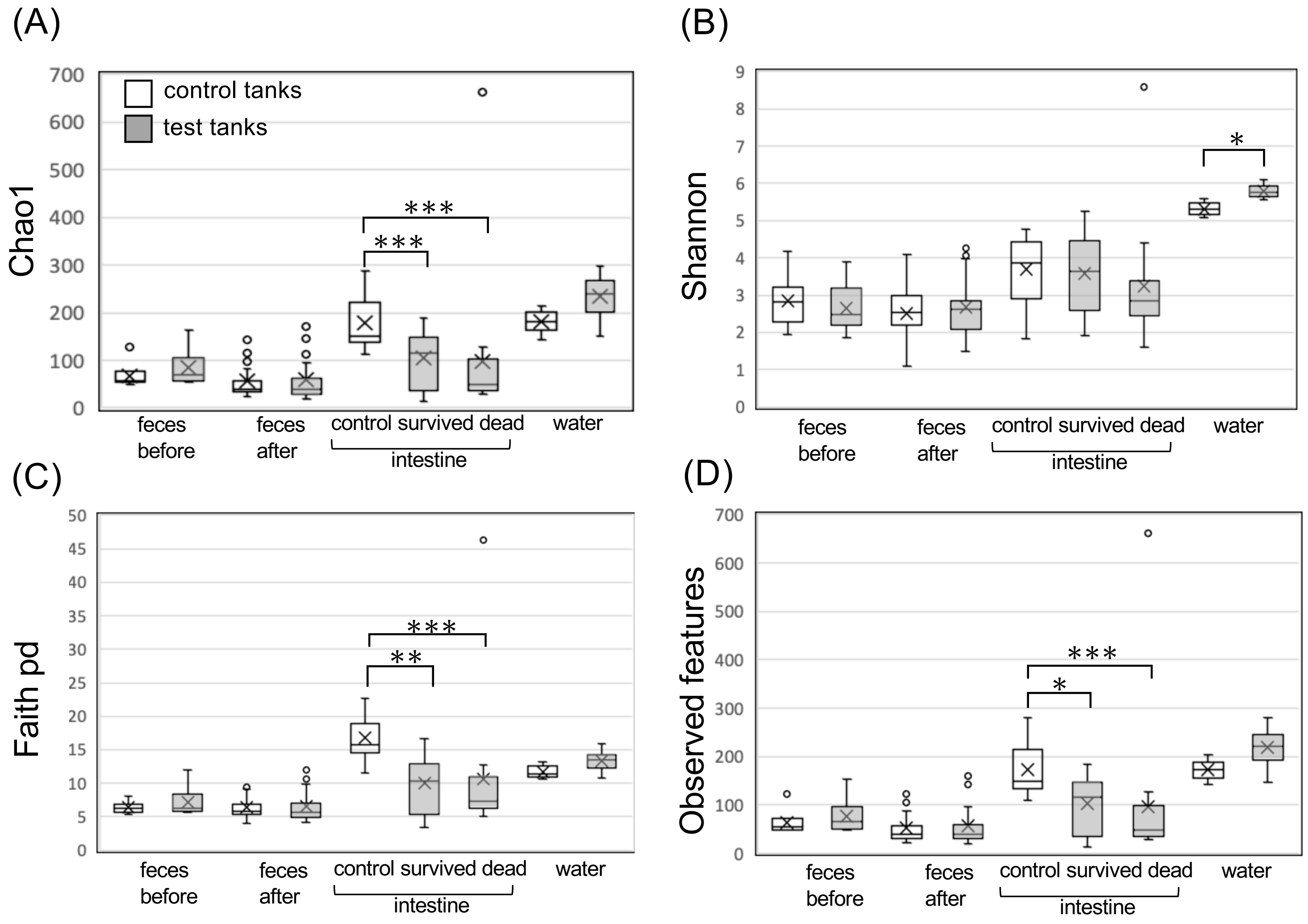

Figure S3. Alpha diversity metrics in feces (before and after the challenge), intestines of *P. altivelis* (control, survived, and dead), and water in control tanks (white) and test tanks (grey). (A) Chao1 index, (B) Shannon-Wiener index, (C) Faith pd, (D) Observed features. *q* values are indicated for samples with significant difference. *: *q* <0.05, **: *q* <0.01 ***: *q* <0.001

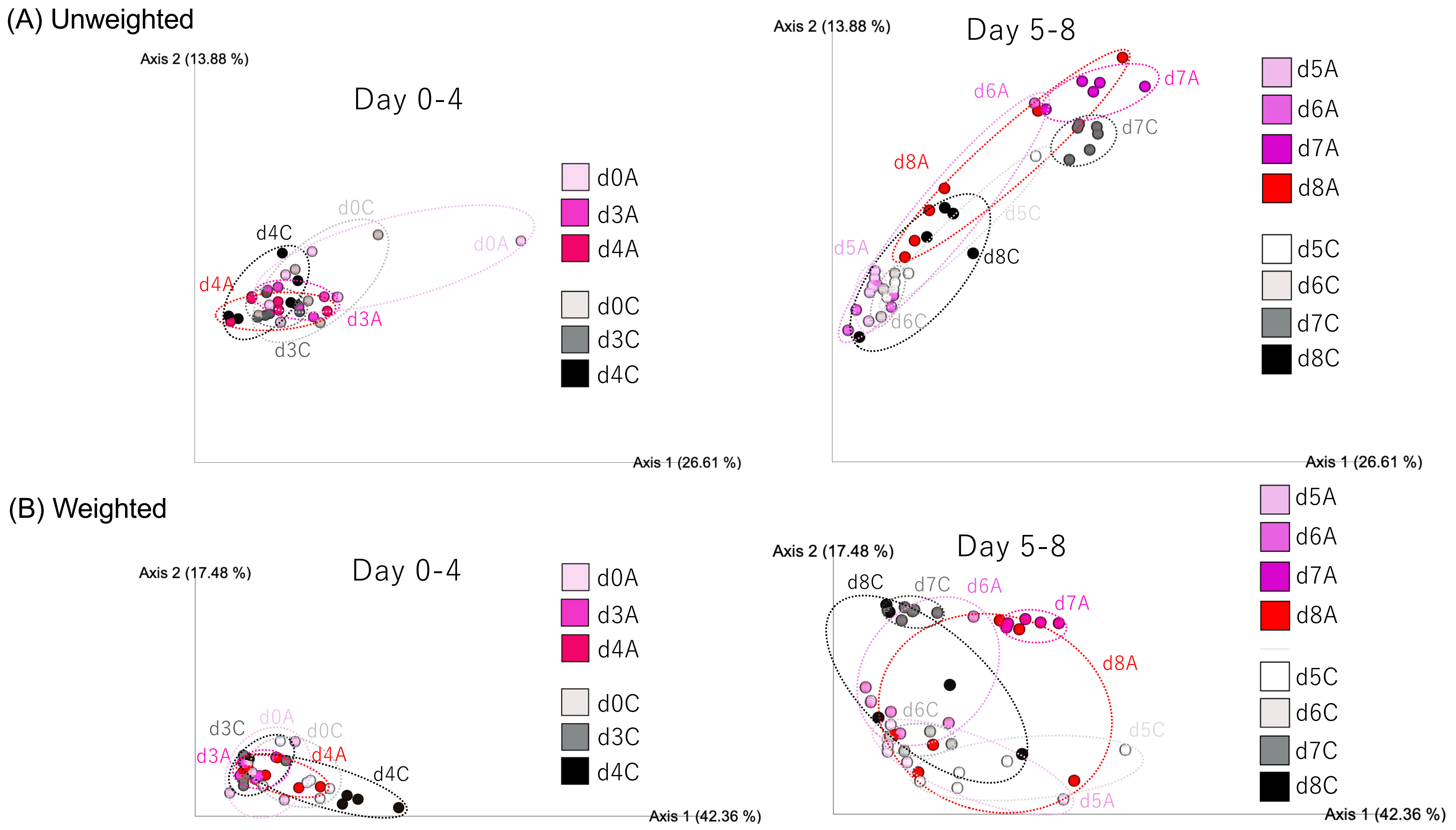

Figure S4. Principle coordinates analysis plots of microbial communities in feces on day 0, 3, 4, 5, 6, 7, and 8. Data are presented according to (A) unweighted and (B) weighted Unifrac.

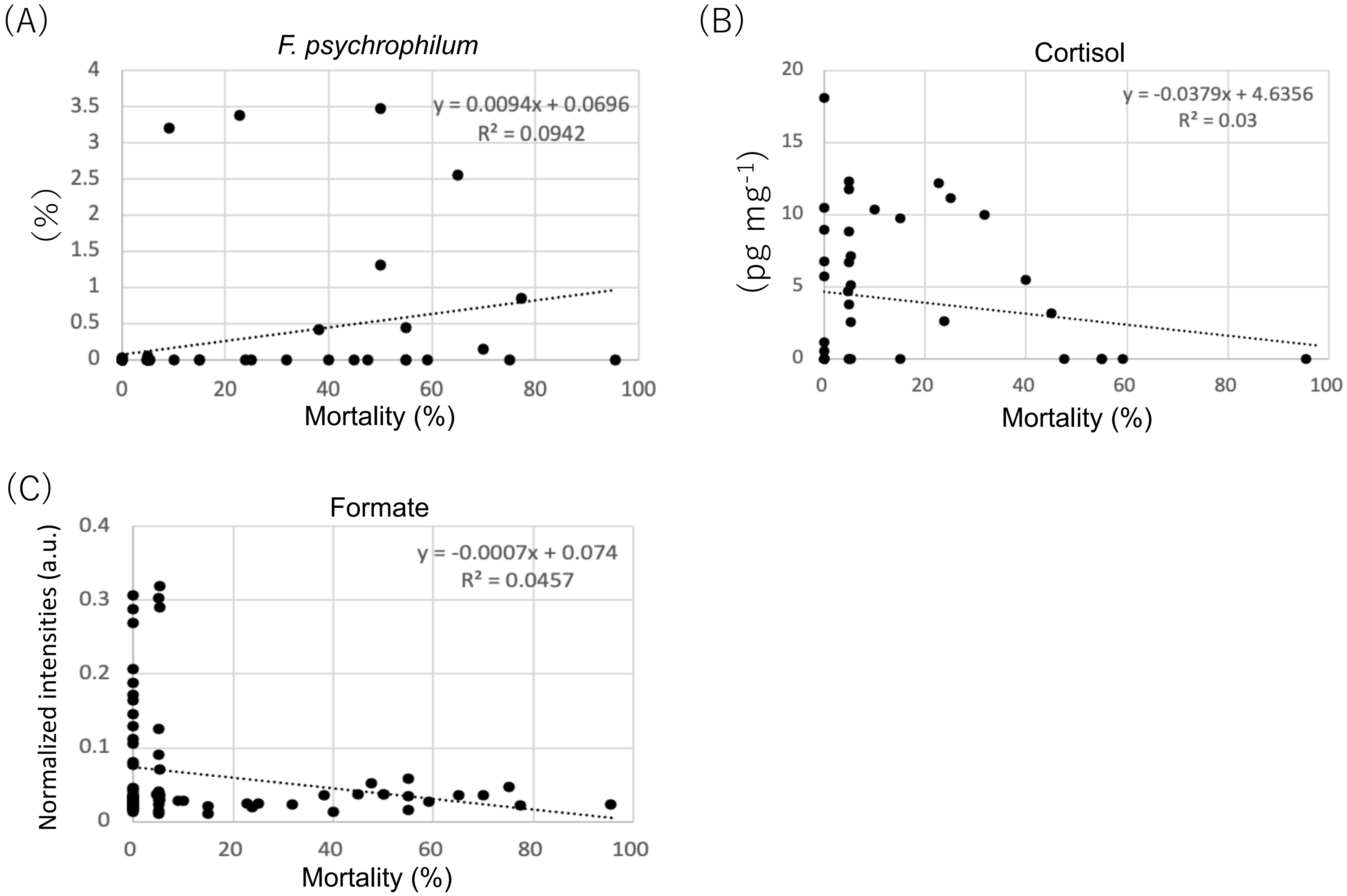

Figure S5. Correlations between mortality and (A) relative abundances of *Flavobacterium psychrophilum*, (B) concentrations of cortisol, and (C) levels of formate.

	acetate	glucose	formate
Acinetobacter	−0.149	0.039	−0.432
Aeromonas	0.084	−0.004	0.343
Klebsiella	−0.249	0.078	−0.151
Enterobacteriaceae group	−0.264	−0.175	0.265
Chryseobacterium	−0.034	−0.270	−0.094
Vogesella	0.306	0.156	0.407
Flectobacillus	0.214	0.199	0.411
Pararheinheimera	0.258	0.197	0.379
Flavobacterium	0.307	0.253	0.150
Vibrio	0.093	0.115	0.250
Shewanella	0.215	−0.122	0.155
Pseudomonas	0.086	−0.073	0.226
Shinella	0.217	0.385	0.173
Cypionkella	0.382	0.321	0.130
Comamonas	0.109	0.085	0.112
Rosenbergiella	−0.010	−0.042	0.117
Deinococcus	0.129	0.269	−0.102
Agrobacterium	−0.075	−0.159	0.369
Rhizobium	−0.088	−0.240	0.023
Burkholderiaceae group	0.316	−0.059	0.228
Hydrogenophaga	0.215	0.165	0.364
Pseudomonas.1	0.194	−0.044	0.125
Emticicia	0.312	0.155	0.383
Acidovorax	0.097	−0.176	0.135
Sphingobium	0.203	−0.040	0.343
Rhodococcus	0.022	0.214	−0.053
Epilithonimonas	0.116	0.253	−0.025
Buttiauxella	−0.028	0.124	−0.193
Rhizobiaceae group	−0.030	−0.055	0.401
Alphaproteobacteria.	0.099	0.234	0.295
Brevundimonas	0.082	−0.241	−0.023
Bosea	0.047	0.123	0.395
Exiguobacterium	0.245	−0.177	−0.138
Pseudomonas.2	0.196	−0.078	0.173
Macrococcus	−0.082	0.143	−0.092
Stenotrophomonas	−0.202	−0.264	−0.165
Aliivibrio	−0.034	0.006	0.443
Fluviicola	0.252	0.269	0.314
Alkanindiges	0.248	0.151	0.122
Sphingopyxis	−0.138	−0.131	0.220
Paenarthrobacter	−0.083	−0.025	0.423
Allorhizobium	0.076	0.187	0.167
Ralstonia	−0.215	0.006	0.136
Novosphingobium	0.034	−0.026	0.321
Deefgea	0.416	0.374	0.028

Figure S6. Spearman's Rank Correlation table between metabolites and relative percentages of bacterial genera.
