# Supplementary material for: Fecal metagenomic and metabolomic analyses reveal non-invasive biomarkers of Flavobacterium psychrophilum infection in ayu (Plecoglossus altivelis)
Source: mSphere. 2024 Jun 17;9(7):e00301-24. doi: 10.1128/msphere.00301-24 (PMC11288038; doi:10.1128/msphere.00301-24)
Supplement: Supplemental text — for Materials and Methods. [file msphere.00301-24-s0002.docx]

**MATERIALS AND METHODS**

**16S rRNA gene sequencing analysis**

After DNA extraction, prokaryotic 16S rRNA gene was amplified by PCR from 125 DNA samples in total using the universal forward primer (Univ515F: 5′- ACACTCTTTCCCTACACGACGCTCTTCCGATCT-GTGCCAGCMGCCGCGGTAA -3′), reverse primer (Univ806R: 5′-GTGACTGGAGTTCAGACGTGTGCTCTTCCGATCT-GGACTACHVGGGTWTCTAAT -3′), and *Ex Taq* HS DNA Polymerase (Takara Bio Inc., Shiga, Japan) with the following conditions: initial denaturation at 94°C for 2 min, denaturation at 94°C for 30 s, annealing at 50°C for 30 s, elongation at 72°C for 30 s, and a final extension at 72°C for 5 min. The PCR cycle numbers were 20. PCR products were purified using AMPure XP (Beckman Coulter, Brea, MA, USA) before a second round of PCR. Purified 16S rRNA genes were sequenced using a MiSeq system (Illumina, San Diego, CA, USA) with a read length of 2×250-bp by Fasmac (Kanagawa, Japan). Raw 16S rRNA gene sequences were analyzed using QIIME 2 ver. 2022.2 (1) and DADA2 (2) to produce ASV sequences. Taxonomic assignment of the non-chimeric ASV sequences was performed using classify-sklearn retained in the Greengenes database (Greengenes2 2022.10) (3). Sequences assigned to chloroplasts and mitochondria were removed. PCoA plots were generated using unweighted (which only considers the presence of taxa) /weighted (which takes into account differences in abundance of taxa) UniFrac distances. Alpha diversity indices were calculated using “qiime diversity alphararefaction” in QIIME 2 (even sampling at 10,917 reads). The differential abundance between groups at the genus level was tested using ANCOM (Analysis of Composition of Microbiomes) (4) with the QIIME2 ANCOM plugin.

**Shotgun metagenomic sequence analysis**

The VAHTS Universal Plus DNA Library Prep Kit for Illumina (Vazyme Biotech Co., Ltd., Nanjing, China) was used for library preparation. The prepared libraries were sequenced using a DNBSEQ G-400 (MGI Tech Co., Ltd., Guangdong, China). Raw reads obtained by sequencing were trimmed using Trimmomatic 0.39 (with the parameters MINLEN:100 ILLUMINACLIP:trim.fa:2:30:10) (5). De novo assembly of the sequences was performed using Megahit v1.2.9 (--k-min 27 --k-max 141 --k-step 12) (6). Sequences from nine samples were co-assembled. Assembled contigs with short length (< 2,500 bp) were removed before the binning and binning was performed using Metabat2 version 2.2.7 (7) with the default parameters. The quality of the bins was assessed using CheckM ver. 1.1.3 (8). Taxonomic classification of the bins was carried out using GTDB-Tk v2.0.0 (GTDB release207; default parameters) (9). The median/mean coverage of the metagenomic bins was calculated using the Metabat2 pipelines (jgi_summarize_bam_contig_depths, default parameters). The relative abundance of metagenomic bins was calculated using coverm 0.4.0 (https://github.com/wwood/CoverM). After the binning, no high-quality bins were assigned to the genera *Acinetobacter* and *Aeromonas*, and unbinned contigs with high abundance were clearly separated from the others (Table S6). To confirm the phylogenetic positions of the unbinned contigs, genomes were compared using the MASH and goANI algorithms with dRep ver. 3.2.0 (10). *Ac. johnsonii* (GCA_003335165.1), *Ac. calcoaceticus* (GCA_000368965.1), and *Ac. pittii* (GCA_003051965.2) were used as reference for *Acinetobacter*, while *Ae. veronii* (GCA_002866885.2) and *Ae. hydrophila* (GCA_000014805.1) were used for *Aeromonas*. Contigs placed close to the genomes of *Aeromonas* and *Acinetobacter* were merged as pangenomes (named Aerom and Acinet, respectively). All bins were annotated using Prokka v1.14.6 (11), GhostKOALA (12), and DRAM software (--use_uniref option with default settings) (13). Biosynthetic gene clusters were identified using antiSMASH ver. 6.1.1 (14).

**REFERENCES**

1. Bolyen E, Rideout JR, Dillon MR, Bokulich NA, Abnet CC, Al-Ghalith GA, Alexander H, Alm EJ, Arumugam M, Asnicar F, Bai Y, Bisanz JE, Bittinger K, Brejnrod A, Brislawn CJ, Brown CT, Callahan BJ, Caraballo-Rodríguez AM, Chase J, Cope EK, Da Silva R, Diener C, Dorrestein PC, Douglas GM, Durall DM, Duvallet C, Edwardson CF, Ernst M, Estaki M, Fouquier J, Gauglitz JM, Gibbons SM, Gibson DL, Gonzalez A, Gorlick K, Guo J, Hillmann B, Holmes S, Holste H, Huttenhower C, Huttley GA, Janssen S, Jarmusch AK, Jiang L, Kaehler BD, Kang KB, Keefe, CR, Keim P, Kelley ST, Knights D, Koester I, Kosciolek T, Kreps J, Langille MGI, Lee J, Ley R, Liu YX, Loftfield E, Lozupone C, Maher M, Marotz C, Martin BD, McDonald D, McIver LJ, Melnik AV, Metcalf JL, Morgan SC, Morton JT, Naimey AT, Navas-Molina JA, Nothias LF, Orchanian SB, Pearson T, Peoples SL, Petras D, Preuss ML, Pruesse E, Rasmussen LB, Rivers A, Robeson MS, Rosenthal P, Segata N, Shaffer M, Shiffer A, Sinha R, Song SJ, Spear JR, Swafford AD, Thompson LR, Torres PJ, Trinh P, Tripathi A, Turnbaugh PJ, Ul-Hasan S, van der Hooft JJJ, Vargas F, V´azquez- Baeza, Y, Vogtmann, E, von Hippel, M, Walters, W, Wan, Y, Wang, M, Warren, J, Weber, KC, Williamson, CHD, Willis, AD, Xu, ZZ, Zaneveld, JR, Zhang, Y, Zhu, Q, Knight, R, Caporaso, JG. 2019. Reproducible, interactive, scalable and extensible microbiome data science using QIIME 2. Nat Biotechnol 37:852–857.
2. Callahan BJ, McMurdie PJ, Rosen MJ, Han AW, Johnson AJA, Holmes SP. 2016 DADA2: high-resolution sample inference from Illumina amplicon data. Nat Methods 13:581–583.
3. DeSantis TZ, Hugenholtz P, Larsen N, Rojas M, Brodie EL, Keller K, Huber T, Dalevi D, Hu P, Andersen GL. 2006. Greengenes, a chimera-checked 16S rRNA gene database and workbench compatible with ARB. Appl Environ Microbiol 72:5069–72.
4. Mandal S, Van Treuren W, White RA, Eggesbø M, Knight R, Peddada SD. 2015. Analysis of composition of microbiomes: a novel method for studying microbial composition. Microb Ecol Health Dis 26:27663.
5. Bolger AM, Lohse M, Usadel B. 2014. Trimmomatic: a flexible trimmer for Illumina sequence data. Bioinformatics 30:2114–2120.
6. Li D, Liu CM, Luo R, Sadakane K, Lam TW. 2015. MEGAHIT: an ultra-fast single-node solution for large and complex metagenomics assembly via succinct de Bruijn graph, Bioinformatics 31:1674–1676.
7. Kang DD, Li F, Kirton E, Thomas A, Egan R, An H, Wang Z. 2019. MetaBAT 2: an adaptive binning algorithm for robust and efficient genome reconstruction from metagenome assemblies. PeerJ 7:e7359.
8. Parks DH, Imelfort M, Skennerton CT, Hugenholtz P, Tyson GW. 2015. CheckM: assessing the quality of microbial genomes recovered from isolates, single cells, and metagenomes. Genome Res 25:1043–1055.
9. Chaumeil P-A, Mussig AJ, Hugenholtz P, Parks DH. 2022. GTDB-Tk v2: memory friendly classification with the genome taxonomy database. Bioinformatics 38:5315–5316.
10. Olm MR, Brown CT, Brooks B, Banfield JF. 2017. dRep: a tool for fast and accurate genomic comparisons that enables improved genome recovery from metagenomes through de-replication. ISME J 11:2864–2868.
11. Seemann T. 2014. Prokka: rapid prokaryotic genome annotation. Bioinformatics 30:2068–2069.
12. Kanehisa M, Sato Y, Morishima K. 2016. BlastKOALA and GhostKOALA: KEGG tools for functional characterization of genome and metagenome sequences. J Mol Biol 428:726–731.
13. Shaffer M, Borton MA, McGivern BB, Zayed AA, La Rosa SL, Solden LM, Liu P, Narrowe AB, Rodríguez-Ramos J, Bolduc B, Gazitúa MC, Daly RA, Smith GJ, Vik DR, Pope PB, Sullivan MB, Roux S, Wrighton KC. 2020. DRAM for distilling microbial metabolism to automate the curation of microbiome function. Nucleic Acids Res 48:8883–8900.
14. Blin K, Shaw S, Kloosterman AM, Charlop-Powers Z, van Wezel GP, Medema MH, Weber T. 2021. antiSMASH 6.0: improving cluster detection and comparison capabilities. Nucleic Acids Res 49:W29–W35.
